# Ohio College of Dental Surgery-Commencement Exercises

**Published:** 1870-04

**Authors:** 


### OHIO COLLEGE OF DENTAL SURGERY—COMMENCEMENT EXERCISES.

THE Ohio College of Dental Surgery held its twenty-fourth annual commencement in the audience room of the college, March 2d.

There was a large attendance to witness the closing exercises of one of the most prosperous years the institution has yet enjoyed. In the audience, too, were many ladies.

The Scriptures were read, and prayer made by the Rev. Dr. Aydelott.

The address of the President of the Board of Trustees is given in the March REGISTER, after the delivery of which the degree of Doctor of Dental Surgery was conferred upon the following young gentlemen: H. K. Lathrop, Jr., Detroit, Michigan; J. M. Porter, Canal Fulton, Ohio; E. C. Moore, Batavia, Ohio; W. T. Martin, Pickens, Mississippi; W. A. Bracy, Raymond, Mississippi; R. J. Weldin, Augusta, Kentucky; Alex. Warner, St. Clair, Michigan; R. F. Ralls, Bath, Kentucky; W. D. Tremper, Cincinnati, Ohio; W. Van Antwerp, Ypsilanti, Michigan.

The President said that it had been the custom of the Faculty, on the graduation of students, to also present them with copies of the Holy Bible. He therefore handed each a neat volume of the Scriptures, at the same time urging them, while making their text books their guides in the practice of their profession, to make this Book of books the guide for the government of their lives. This somewhat unusual practice in graduation at institutions of science, was the more noticeable inasmuch as there was a vein of religious sentiment running through all the exercises, and in almost all that was said a full recognition of the plan of salvation through a crucified Savior.

After the conferring of the degrees, the annual address was delivered by Dr. W. H. Morgan, of Nashville, Tenn., to which a very spirited and well-timed reply was made by H. K. Lathrop, of Detroit, both of which will be found in the March number of the REGISTER.

The exercises were interspersed by most excellent music.

In addition to the regular graduates, the Board of Trustees conferred the Honorary degree upon E. C. Sloan, Ironton, Ohio; S. M. Cummings, Elkhart, Ind; W. M. Herriott, Zanesville, O.; S. Clippenger, Toledo, O.; B. F. Rosson, Troy, O., and E. V. Burt, Lafayette, Ind.

This action of the Board was based upon the indisputable merit of the recipients. T.

